# Jejunal Loop-Preserving and Mesentery-Sparing Reconstruction after Pancreaticoduodenectomy in a Patient with Prior Total Gastrectomy

**DOI:** 10.70352/scrj.cr.26-0477

**Published:** 2026-09-30

**Authors:** Rena Mototani, Keiichi Akahoshi, Takaki Furuyama, Daisuke Asano, Yoshiya Ishikawa, Hiroki Ueda, Eriko Katsuta, Daisuke Ban

**Affiliations:** 1 Department of Hepatobiliary and Pancreatic Surgery, Institute of Science Tokyo, Tokyo, Japan; 2 Department of Professional Development, Institute of Science Tokyo, Tokyo, Japan

**Keywords:** total gastrectomy, pancreaticoduodenectomy, reconstruction, jejunal loop-preserving, mesentery-sparing

## Abstract

**INTRODUCTION:**

Pancreaticoduodenectomy (PD) after a total gastrectomy is technically challenging because the proximal jejunum has already been utilized for an esophagojejunostomy. Consequently, reconstruction after PD requires the use of a more distal jejunal segment, and the optimal reconstruction strategy remains controversial. We report a case of a jejunal loop-preserving and mesentery-sparing reconstruction after PD in a patient with a prior total gastrectomy.

**CASE PRESENTATION:**

A 70-year-old man with a history of Roux-en-Y total gastrectomy for gastric cancer presented with obstructive jaundice. Imaging studies demonstrated a periampullary tumor with suspected regional lymph node metastasis, and ampullary carcinoma was diagnosed. A PD was performed through an upper midline laparotomy. Following the resection of the pancreatic head and proximal jejunum, reconstruction was achieved using the pre-existing Roux limb without additional mesenteric division. Pancreaticojejunostomy and hepaticojejunostomy were performed using a distal jejunal segment, followed by a side-to-side jejunojejunostomy that preserved jejunal continuity and mesenteric integrity. The operative time was 459 minutes, and blood loss was 603 mL. The postoperative course was uneventful, and the patient was discharged on POD 21. Histopathological examination confirmed adenocarcinoma of the ampulla of Vater (pT3bN1M0, Stage IIIA) with negative surgical margins.

**CONCLUSIONS:**

This jejunal loop-preserving and mesentery-sparing reconstruction avoided additional jejunal transection and mesenteric dissection while maintaining the continuity of the jejunal loop. The technique may represent a simple and anatomically reasonable alternative to conventional double Roux-en-Y reconstruction in selected patients undergoing PD after a total gastrectomy.

## Abbreviations

CA19-9
: carbohydrate antigen 19-9
CEA
: carcinoembryonic antigen
FDG
: fluorodeoxyglucose
PD
: pancreaticoduodenectomy
SUVmax
: maximum standardized uptake value

## INTRODUCTION

PD is a complex surgical procedure,^1)^ and reconstruction after PD becomes particularly challenging in patients with prior gastrectomy due to altered gastrointestinal anatomy. Although PD following a gastrectomy has been considered relatively rare, its incidence is expected to increase with improved long-term survival after gastric cancer and the rising prevalence of pancreatic and bile duct malignancies.^2)^

Various types of reconstruction are performed after a gastrectomy, including Billroth I, Billroth II, and Roux-en-Y (R-Y) reconstruction.^3)^ In patients with a prior Billroth I reconstruction, PD can typically be carried out using steps comparable to those of conventional PD.^4)^ However, in patients who have undergone a total gastrectomy with Roux-en-Y reconstruction, both resection and reconstruction become technically demanding.

In standard PD, the proximal jejunum is typically used for pancreaticojejunostomy and hepaticojejunostomy, most commonly in a modified Child reconstruction.^2)^ Similarly, in a total gastrectomy with Roux-en-Y reconstruction, the proximal jejunum is used for an esophagojejunostomy.^5)^ Therefore, when PD is performed after a total gastrectomy, reconstruction must be performed using a more distal jejunal segment while preserving an adequate vascular supply.

In such cases, a double Roux-en-Y reconstruction with the creation of an additional jejunal limb is commonly used.^4)^ However, mobilization of the jejunal limb requires mesenteric dissection, which may compromise mesenteric vessels and increase the risk of ischemia or venous congestion of the lifted jejunum.^6)^ Particular concerns include the preservation of mesenteric blood flow and the avoidance of excessive tension on the reconstructed jejunal limb. Several reconstruction strategies have been reported for PD after a total gastrectomy, but the optimal approach remains controversial. The recently described uncut Roux-en-Y technique preserves jejunal continuity while functionally occluding the afferent jejunal limb using a stapler. Although its application after a total gastrectomy was illustrated, the reported clinical series included only patients with a previous radical subtotal gastrectomy.^7)^

Herein, we present a jejunal loop-preserving and mesentery-sparing reconstruction that maintains a patent, continuous jejunal loop without functional occlusion or additional mesenteric division in a patient undergoing PD after a Roux-en-Y total gastrectomy.

## CASE PRESENTATION

### Patient information and diagnosis

A 70-year-old man with a history of Roux-en-Y total gastrectomy, cholecystectomy, and splenectomy for gastric cancer 16 years previously presented with obstructive jaundice. Laboratory examination demonstrated elevated total bilirubin (29.2 mg/dL), CEA (3.5 ng/mL), and CA19-9 (4641 U/mL). Contrast-enhanced CT revealed a 26 × 25-mm periampullary mass associated with biliary dilatation and mild pancreatic duct dilatation (**Fig. 1A**). Gadolinium ethoxybenzyl diethylenetriamine pentaacetic acid-enhanced MRI confirmed a 29-mm periampullary mass without evidence of liver metastasis.

**Fig. 1:**
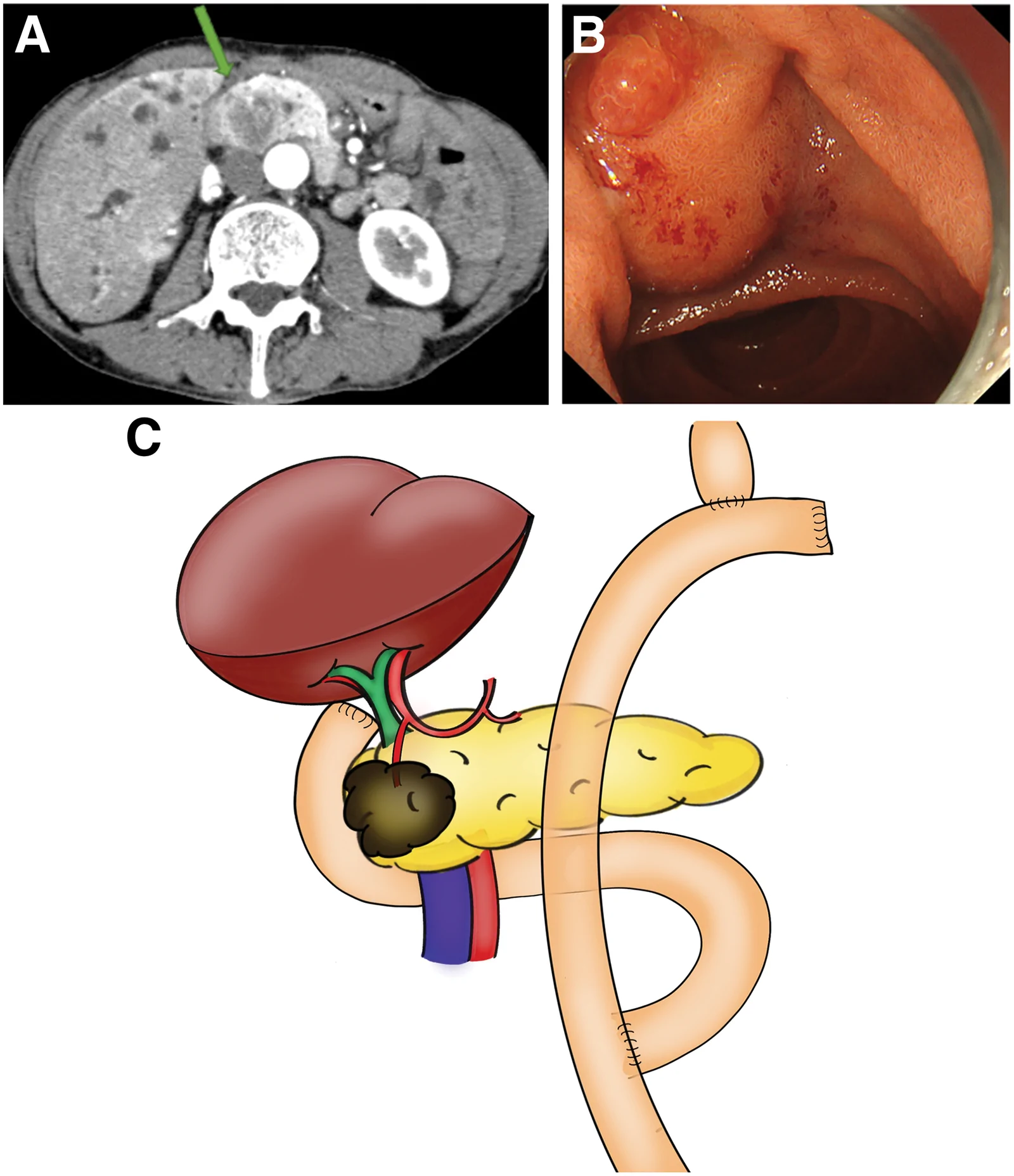
Preoperative findings and anatomy after previous total gastrectomy. (**A**) Contrast-enhanced CT demonstrating a periampullary mass (arrow) associated with biliary dilatation. (**B**) Endoscopic findings showing a nonexposed ampullary tumor. (**C**) Schematic illustration of the anatomy after Roux-en-Y total gastrectomy. The proximal jejunal limb had already been utilized for esophagojejunostomy, necessitating the use of a more distal jejunal segment for reconstruction after PD. PD, pancreaticoduodenectomy

Percutaneous transhepatic cholangiodrainage was performed, and bile cytology was classified as class III (suspicious for malignancy). Endoscopic retrograde cholangiopancreatography was technically challenging because of the previous total gastrectomy; however, the appearance of the papilla was suggestive of a nonexposed ampullary carcinoma (**Fig. 1B**). PET demonstrated increased FDG uptake in the periampullary lesion (SUVmax 7.3) and a regional lymph node at the hepatic hilum (SUVmax 3.8), raising suspicion of regional lymph node metastasis. No distant metastatic lesions were identified.

Based on these findings, the patient was diagnosed with ampullary carcinoma (cT3bN1M0, Stage IIB), and PD was planned. Because the patient had previously undergone Roux-en-Y total gastrectomy, reconstruction after resection required special consideration.

### Surgical procedure

The anatomy after the previous Roux-en-Y total gastrectomy is shown in **Fig. 1C**. Because the proximal jejunum had already been used for an esophagojejunostomy, reconstruction after PD required a more distal jejunal segment.

PD was performed via an upper midline laparotomy. No evidence of peritoneal dissemination or distant metastasis was observed. A firm 4-cm mass was palpable in the pancreatic head, although no tumor was visibly apparent on the pancreatic surface. The planned resection line is shown in **Fig. 2A**. After mobilization of the pancreatic head from the retroperitoneum, the superior mesenteric vein was exposed along the inferior border of the pancreatic body. The common hepatic artery, proper hepatic artery, and gastroduodenal artery were encircled at the superior border of the pancreas, and the pancreatic neck was taped over the portal vein (**Fig. 3A**). The proximal jejunum was then divided at the level of the original jejunojejunostomy using a linear stapler. Pancreatic transection and bile duct division were subsequently performed, and the specimen was removed en bloc (**Fig. 2B**). The original Roux limb measured approximately 40 cm from the esophagojejunostomy to the original jejunojejunostomy. A window was created in the avascular right side of the transverse mesocolon, through which the distal jejunum was elevated in a retrocolic fashion without mesenteric division. The new pancreaticojejunostomy was located approximately 60 cm distal to the esophagojejunostomy. The pancreaticojejunostomy was performed using the modified Blumgart technique, followed by a hepaticojejunostomy 5–10 cm distal to the pancreaticojejunostomy. The elevated jejunal limb reached both anastomotic sites without excessive tension, and the mesenteric orientation was carefully confirmed to avoid twisting. Jejunal perfusion was assessed intraoperatively by confirming satisfactory bowel color and mesenteric arterial pulsation, together with the absence of bowel or mesenteric edema suggestive of venous congestion. Indocyanine green fluorescence imaging was not used. Subsequently, a side-to-side jejunojejunostomy was created between the site of the original jejunojejunostomy and the jejunum approximately 30 cm distal to the hepaticojejunostomy, thereby preserving both jejunal continuity and mesenteric integrity (**Figs. 2C** and **3B**). After reconstruction, food passed through the pre-existing Roux limb from the esophagojejunostomy to the new side-to-side jejunojejunostomy and then into the distal jejunum, whereas pancreatic juice and bile drained from the pancreaticojejunostomy and hepaticojejunostomy, respectively, and joined the alimentary stream at the new jejunojejunostomy. The operative time was 459 minutes, and the estimated blood loss was 603 mL. No intraoperative complications occurred.

**Fig. 2:**
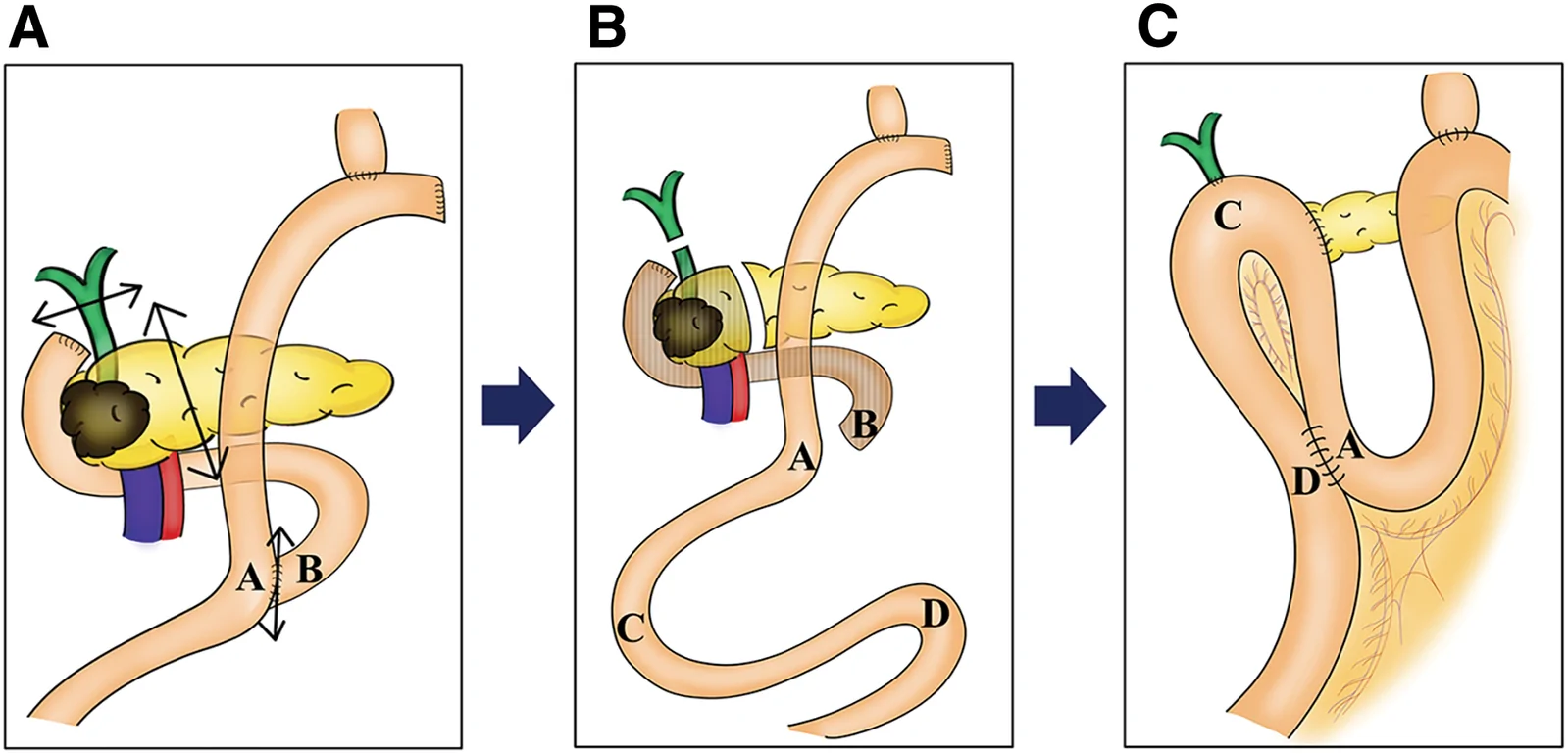
Schematic illustration of the present jejunal loop-preserving and mesentery-sparing reconstruction. (**A**) Planned resection line for PD. The proximal jejunum was divided between points A and B. (**B**) Anatomy after resection of the pancreatic head and proximal jejunum. The shaded area indicates the resected specimen. (**C**) Completed reconstruction. Segment C, located distal to the original jejunojejunostomy (point A) of the previous Roux-en-Y reconstruction, was used for hepaticojejunostomy and pancreaticojejunostomy. Subsequently, point A was anastomosed to point D by side-to-side jejunojejunostomy, creating the alimentary passage while preserving jejunal continuity and mesenteric vasculature without additional mesenteric division. PD, pancreaticoduodenectomy

**Fig. 3:**
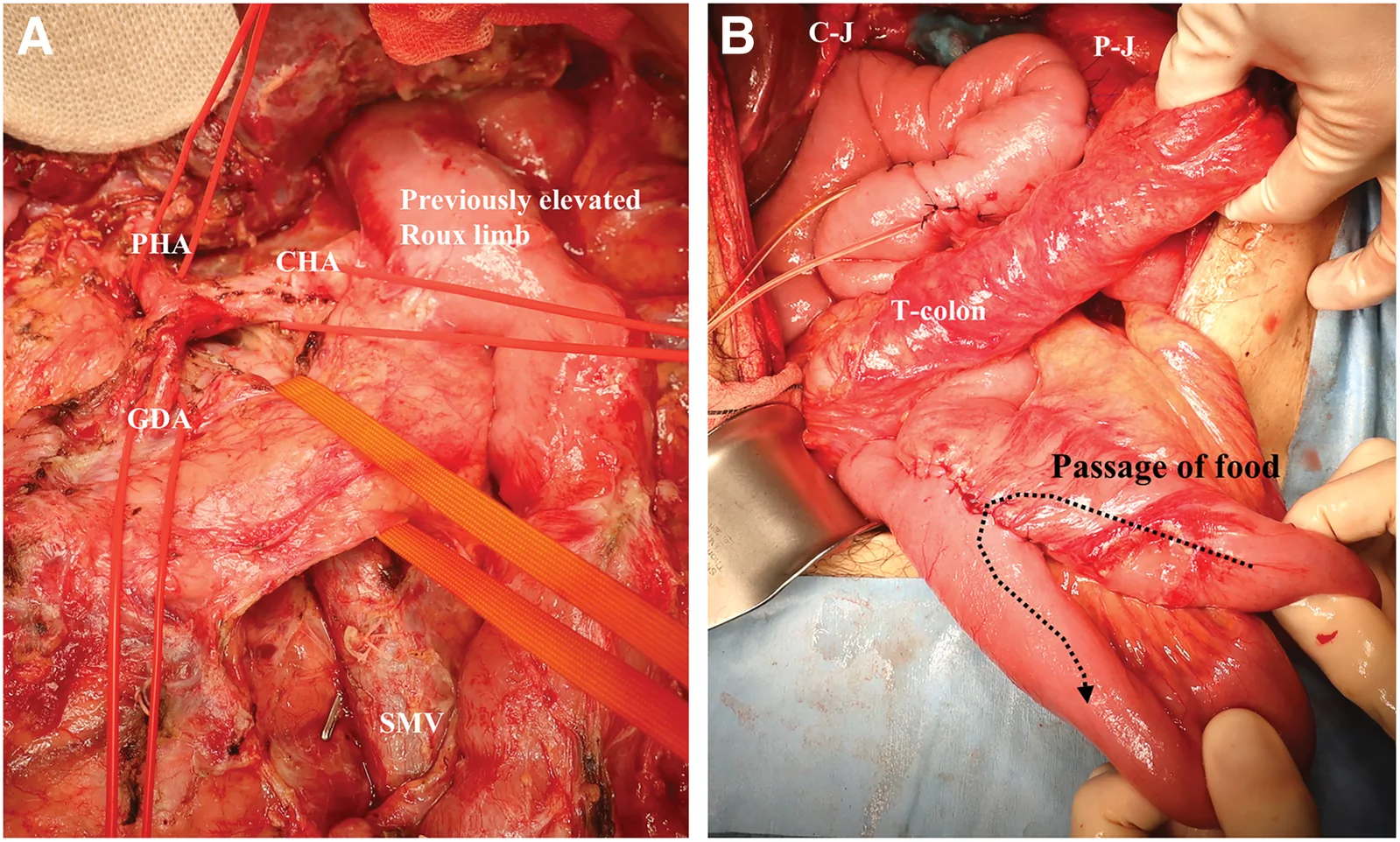
Intraoperative findings. (**A**) Operative view before resection showing taping of the pancreatic neck over the PV and encirclement of major vascular structures. The previously elevated Roux limb is preserved. (**B**) Completed reconstruction demonstrating preservation of the continuous jejunal loop. The arrow indicates the expected passage of food through the reconstructed jejunum. CHA, common hepatic artery; C-J, choledochojejunostomy; GDA, gastroduodenal artery; PHA, proper hepatic artery; P-J, pancreaticojejunostomy; PV, portal vein; SMV, superior mesenteric vein; T-colon, transverse colon

### Pathological findings

A histopathological examination revealed an adenocarcinoma of the ampulla of Vater measuring 45 mm in diameter. The final pathological staging was pT3bN1M0 (Stage IIIA) according to the UICC TNM 8th edition classification. The surgical margins were negative (R0 resection).

### Follow-up

The postoperative course was uneventful. Drain amylase levels were not elevated postoperatively, and no clinically relevant postoperative pancreatic fistula developed. No postoperative complications of Clavien–Dindo grade I or higher were observed. The patient was discharged on POD 21.

Fourteen months after surgery, follow-up CT revealed pulmonary metastasis, and chemotherapy with gemcitabine and cisplatin was initiated. Durvalumab was added 21 months after surgery. Despite systemic treatment, the metastatic disease progressed, and the patient died of bronchopneumonia 30 months after surgery.

## DISCUSSION

PD after a total gastrectomy with Roux-en-Y reconstruction is technically challenging due to the altered gastrointestinal anatomy.^2)^ In particular, the proximal jejunum is already used for an esophagojejunostomy, necessitating a more distal jejunal segment for pancreatic and biliary reconstruction. As a result, the optimal reconstruction strategy in this setting remains controversial.

Previous reports of PD after a total gastrectomy have mainly described 2 reconstruction strategies: reuse of the previous Y limb for pancreatic and biliary reconstruction, or creation of a new Roux limb in a double Roux-en-Y configuration (**Supplementary Table 1**).^4,6,8–11)^ Reuse of the previous Y limb can avoid the creation of an additional jejunal limb; however, a short residual afferent limb has been associated with afferent loop syndrome and recurrent cholangitis.^4,8)^ In contrast, the creation of a new Roux limb provides sufficient length for tension-free pancreatic and biliary reconstruction, but requires additional jejunal division and mesenteric mobilization (**Supplementary Fig. 1**).^4,6,9–11)^

The present reconstruction differs from both strategies. Rather than reusing the pre-existing Y limb for pancreatic and biliary reconstruction or creating an additional divided Roux limb, the distal jejunum is elevated for pancreaticojejunostomy and hepaticojejunostomy without mesenteric division, and alimentary continuity is restored by a side-to-side jejunojejunostomy within the same continuous jejunal loop. Thus, jejunal continuity and the native mesenteric vasculature are preserved without additional jejunal division or mesenteric dissection (**Table 1** and **Fig. 2C**).

**Table 1:** Conceptual differences between conventional double Roux-en-Y reconstruction and the present mesentery-sparing reconstruction

Feature	Double Roux-en-Y reconstruction (conventional method)	Mesenteric-sparing reconstruction (present method)
Jejunal division	Required	Not required
Mesenteric dissection	Required	Not required
Jejunal continuity	Disrupted (divided limbs)	Preserved (single continuous loop)
Blood supply to jejunal limb	Potentially compromised	Preserved

This table summarizes the principal anatomical and technical differences between the conventional double Roux-en-Y reconstruction and the present reconstruction strategy.

The present method preserves jejunal continuity and native mesenteric blood supply without additional jejunal division or mesenteric dissection.

A conceptually related uncut Roux-en-Y reconstruction has recently been reported after a previous radical gastrectomy.^7)^ Although the authors illustrated its potential configuration after a total gastrectomy, all patients in their reported clinical series had undergone a radical subtotal gastrectomy. In the uncut Roux-en-Y technique, anatomical jejunal continuity is preserved, but the afferent jejunal limb is functionally occluded using a stapler to prevent food passage toward the pancreatic and biliary anastomoses. In contrast, the present reconstruction maintains a fully patent, continuous jejunal loop without functional occlusion or interruption of luminal flow and requires no additional mesenteric division. These features distinguish the present reconstruction from the previously described uncut Roux-en-Y concept.

A key technical point of the present reconstruction is the tension-free elevation of the preserved jejunal loop while maintaining the native orientation of the mesentery. Because the mesentery is not divided, careful attention is required to avoid twisting or kinking of the jejunal loop during retrocolic elevation. Before performing the pancreaticojejunostomy and hepaticojejunostomy, the limb should reach the anastomotic sites without excessive tension, and adequate perfusion should be confirmed by assessing bowel color, mesenteric arterial pulsation, and the absence of bowel or mesenteric edema suggestive of venous congestion. Based on these technical requirements, the present reconstruction may be suitable when the available jejunal loop has sufficient length to reach the pancreatic and biliary anastomotic sites without excessive tension, and mesenteric perfusion is satisfactorily preserved, and adhesions do not prevent safe elevation of the jejunum without mesenteric division. Conversely, this approach may be less suitable when the available jejunal segment is too short, mesenteric vascularity is compromised, or dense adhesions or mesenteric distortion prevents tension-free elevation without twisting or kinking. In such situations, the creation of a new Roux limb or another reconstruction strategy should be considered. Because the present report describes a single case, these conditions should be regarded as practical considerations for patient selection rather than established indications or contraindications.

Another potential advantage of this configuration is its applicability to postoperative endoscopic management. In cases requiring endoscopic intervention after PD, such as a pancreatic fistula or biliary stone formation,^12,13)^ the presence of multiple accessible routes to the pancreaticojejunal and hepaticojejunostomy sites may theoretically facilitate endoscopic access. However, this potential advantage was not evaluated in the present case, and the actual feasibility of postoperative endoscopic intervention remains uncertain.

Potential disadvantages of the present configuration should also be considered. Because the alimentary stream passes in close proximity to the pancreaticojejunostomy and hepaticojejunostomy, reflux of enteric contents into the biliopancreatic limb may theoretically occur and could contribute to afferent limb stasis or cholangitis. In addition, preserving a continuous jejunal loop may increase the risk of angulation or kinking if the limb is not positioned without tension or mesenteric twisting. None of these complications occurred in the present case; however, their incidence and clinical significance cannot be determined from a single-case experience.

This report has several limitations. First, this is a single-case experience, and the generalizability of the technique remains uncertain. Second, the theoretical advantages regarding intestinal flow dynamics and postoperative endoscopic accessibility were not objectively evaluated. Further studies are required to determine the safety, reproducibility, and long-term clinical benefits of this reconstruction method.

## CONCLUSIONS

In conclusion, we present a jejunal loop-preserving and mesentery-sparing reconstruction technique after PD in a patient with prior total gastrectomy. This approach may represent a simple and anatomically reasonable alternative to conventional reconstruction methods in selected patients.

## SUPPLEMENTARY MATERIALS

Figure 1 Conventional double Roux-en-Y reconstruction A conventional double Roux-en-Y reconstruction after a pancreaticoduodenectomy in a patient with a prior total gastrectomy.

Table 1 Published reconstruction strategies and the present case for pancreaticoduodenectomy after total gastrectomy Abbreviations: EJ, esophagojejunostomy; HJ, hepaticojejunostomy; JJ, jejunojejunostomy; NR, not reported; PD, pancreaticoduodenectomy; PJ, pancreaticojejunostomy; TG, total gastrectomy.
Notes: Only reported cases of pancreaticoduodenectomy after a total gastrectomy were included. For studies including various types of gastrectomy, only cases after a total gastrectomy were extracted when applicable.

## DECLARATIONS

### Availability of data and materials

All relevant clinical data are included within the article.
