# Supplementary material for: Jejunal Loop-Preserving and Mesentery-Sparing Reconstruction after Pancreaticoduodenectomy in a Patient with Prior Total Gastrectomy
Source: Surg Case Rep. 2026 Sep 30;12(1):26-0477. doi: 10.70352/scrj.cr.26-0477 (PMC13634787; doi:10.70352/scrj.cr.26-0477)
Supplement: Supplementary file 1 [file scr-12-01-26-0477-s002.pdf]

Supplemental Table 1. Published reconstruction strategies and the present case for pancreaticoduodenectomy after total gastrectomy

Author, year	No. of cases	Reconstruction strategy	Jejunal limb used for PJ/HJ	Reported limb length or key anastomotic distances (cm)	Postoperative complications	Afferent loop syndrome	Notes / remarks	Reference No.
Yokoyama et al., 2014	1	Previous Y limb reconstruction	Previous afferent limb from TG	15–20 cm afferent limb	Recurrent cholangitis	Yes	• Previous TG afferent limb used for PJ/HJ after PD.<br>• Duodenal portion of previous afferent loop resected, leaving a short 15–20 cm afferent limb.<br>• Recurrent cholangitis due to afferent loop syndrome (ALS).<br>• HJ subsequently was re-operated using a new 40-cm Y limb in a double Roux-en-Y configuration.<br>• Cholangitis resolved after reconstruction.	8)
Lee et al., 2015	2	Double Roux-en-Y reconstruction	New distal jejunal limb	40 cm from HJ to new distal JJ	None	No	• The authors considered preservation of the previous EJ with a new Roux-en-Y reconstruction to be a safe and simple option after TG.	9)
Yokoyama et al., 2017	3	Double Roux-en-Y reconstruction	New Roux limb using jejunum distal to the previous JJ	NR	Pancreatic fistula (1 case)	No	• No cases of afferent loop syndrome were observed in this group.	10)
Kawamoto et al., 2017	3	Double Roux-en-Y reconstruction	New Roux limb using jejunum distal to the previous JJ	50 cm in 2 cases; 60 cm in 1 case (PJ–JJ distance)	None	No	• The authors recommend avoiding use of the remnant afferent jejunum for PJ/HJ when it is <50 cm because of ALS risk.	6)
Lee et al., 2017	7	Double Roux-en-Y reconstruction	New jejunal limb created from distal jejunum	The new pancreaticobiliary limb was designed to have sufficient length of 40–50 cm.	Cholangitis (1 case)	NR	• The authors specifically emphasize creating a sufficiently long new pancreaticobiliary limb to avoid tension and postoperative complications.	11)
Jeon et al., 2022	1	Double Roux-en-Y reconstruction	New jejunal limb	2 cm stump left from previous jejunojejunostomy; new Roux-en-Y limb length NR	None	No	• Remaining jejunum distal to the Treitz ligament was removed en bloc with the PD specimen.<br>• A 2-cm stump from the previous jejunojejunostomy was left.<br>• New Roux-en-Y reconstruction was performed.	4)
Jeon et al., 2022	4	Previous Y limb reconstruction	Remaining jejunum distal to Treitz ligament	NR	Chyle leakage (2 cases); superior mesenteric vein thrombosis (1 case)	No	• Remaining jejunum distal to the Treitz ligament was preserved during PD.<br>• The preserved jejunum was used for both PJ and HJ.<br>• Authors favored using the remaining jejunum when it was sufficiently long, citing shorter operative time and avoidance of a new jejunal anastomosis.	4)
Present case	1	Jejunal loop-preserving and mesentery-sparing reconstruction	Jejunum distal to the original jejunojejunostomy after division of the previous Y-limb	EJ–original JJ: approximately 40 cm; EJ–PJ/HJ: approximately 60 cm; PJ–HJ: 5–10 cm; HJ–new JJ: approximately 30 cm	None	No	• No jejunal division or mesenteric dissection was performed. The native mesenteric vasculature was preserved.	Present report

Abbreviations: EJ, esophagojejunostomy; JJ, jejunojejunostomy; PJ, pancreaticojejunostomy; HJ, hepaticojejunostomy; PD, pancreaticoduodenectomy; TG, total gastrectomy; NR, not reported.

Notes: Only reported cases of pancreaticoduodenectomy after total gastrectomy were included. For studies including various types of gastrectomy, only cases after total gastrectomy were extracted when applicable.
